# Illinois State Dental Society

**Published:** 1887-08

**Authors:** E. E. Cady


### ILLINOIS STATE DENTAL SOCIETY.

Reported for the Independent Practitioner by E. E. Cady, D. D. S.

(Continued from page 376.)

### THURSDAY MORNING SESSION.

This was devoted to clinics and the exhibition of instruments, etc. Dr. C. F. Matteson, the supervisor, reported the successful operations performed at previous meetings for members present. Dr. Louis Ottofy implanted two teeth, for different patients, with every promise of success. Dr. W. B. Ames demonstrated the use of electricity in the treatment of pyorrhœa alveolaris, and succeeded in convincing many who witnessed his clinic that whatever the result, his theory is at least plausible. Dr. J. A. W. Davis exhibited an electrical apparatus for the painless extraction of teeth, but operations illustrating its use were not uniformly satisfactory.

### THURSDAY AFTERNOON SESSION.

Dr. Black was called upon for his report on inoculation, and said: In gathering material from the mouth it is best to use a strong platinum wire, melted into a glass handle, with the end flattened and bent into a loop. This is sterilized by being brought to a glow in a Bunsen flame, and when cool is touched to the mucous membrane of that part of the mouth from which we wish to make a plant. Having done this, a tube of broth is quickly uncorked, the wire thrust in, the tube shaken and the cork replaced, observing care lest the latter, when removed from the tube, does not come in contact with anything. The tube is now replaced in the incubator, where the growth will develop in twenty-four hours. From this, plants may be made to dry plates for separating the species, in the following manner: Prepare four or six ordinary glass slides by cleansing thoroughly and heating over a Bunsen burner sufficiently to scorch cotton, and allow them to cool. Two or three gelatine tubes are then warmed sufficiently to render the gelatine limpid. The first of these, marked No. 1, is planted by dipping the collecting wire into broth-culture and transferring a drop to the tube, which is then thoroughly shaken to distribute the microbes throughout the mass of gelatine. From this gelatine, tube No. 2 is inoculated in the same manner. From No. 1 a few drops are poured on two of the prepared plates, and distributed evenly over the surface with the previously-heated platinum wire. From gelatine tube No. 2, two more glass plates are prepared in the same manner.

These are now placed in the moist chamber and allowed to remain one or two days for colonies to develop. Sometimes it will be found necessary to carry attenuation to a third tube, in order that colonies may be sufficiently distributed on the plate for easy work in removal. At the end of twenty-four, thirty-six or forty-eight hours the plates may be examined by the microscope, and when the colonies are sufficiently developed they should be transferred to broth-tubes. This is a very important and delicate procedure. A platinum wire of sufficient stiffness is melted into a glass handle, the point reduced and bent at a right angle to form a short hook. This is sterilized in the usual manner. A colony is selected and the point of the wire passed under the lens and over the colony. With the eye at the eye-piece of the microscope the movements of the wire are directed, and the point is brought into the centre of the colony. This rod is then quickly transferred to the broth-tube, with the gelatine and microbes adhering to its point, and is given a shake, removed, and the tubes closed and placed in the incubator. This will give a growth of microbes, all of which have developed from a single cell.

This operation is repeated until each variety appearing upon the plate is transferred to separate broth-tubes. These will develop in different lengths of time, according to habits of the various species, and their growth in broth may be studied.

To obtain growth in gelatine, I pursue the following method: Remove the rubber cover from the mouth of a gelatine tube, and start the cork out by twisting, holding the tube bottom end up between the thumb and finger. Now place the tooth-growth between the same thumb and finger, with the top uppermost. A fine, straight platinum wire (prepared and sterilized as just mentioned) is used, and while cooling the corks from both tubes are removed and held between the fingers. The wire is passed into the tooth-growth, and then pricked through to the bottom of the gelatine. It is again dipped into the tooth-growth, passed into the gelatine tube, laid upon the sloping surface and withdrawn. The corks are now replaced, the mouth of the gelatine tube again covered with rubber, and it is laid away in an inclined position to develop. The anærobic microbe will grow about as well within the gelatine as on the surface, but the strictly ærobic will not make much development when excluded from free oxygen.

In making those cultures many "weeds" will be found interspersed with the growths of the mouth. We are passing about through the atmosphere and breathing it in, and the microbes afloat are continually lodging in our saliva. This latter is a good culture fluid, therefore we collect a great variety. The most of these are non-residents, and do not properly belong in the mouth. A few varieties, however, are found there almost continually, and these we may properly call residents. The former we may denominate weeds, but the latter seem indigenous to the soil, and are continually pushing out the intruders. Of course, in making cultivations from the mouth we may, in time, separate a great number of species of microbes, but we can only designate those habitually found in the mouth after repeated cultivations. The rest must be regarded as strangers.

OPERATIVE DENTISTRY.

Dr. Noyes, who opened the discussion, spoke as follows: My attention has been specially directed to the cervical borders of fillings in the last few months, with the result that I have come to the conclusion that this point is often neglected. The proper finishing of cervical margins is of the greatest importance, but does not receive proper attention oftentimes, because at the end of a long operation both the patient and operator are tired and nervous. In filling a tooth the first important point is to have the edges of the cavity carefully smoothed before inserting the filling. The next point of great importance is plenty of space—separation. It often happens that while we may have sufficient room to fill a tooth properly, there is not enough for finishing. The next point is complete exposure to the eye of the edge of the filling near or above the gum. An orange-wood wedge may be used for securing this, the painfulness usually resulting therefrom being controlled by the application of cocaine to the gums. In using disks, great care is necessary, the tendency being to trim away the lower third of the filling too much, the upper not enough.

Do not use pieces of gold so large as to obscure the view or endanger the certainty of thorough packing. First and second permanent molars, soon after eruption, may sometimes be filled with gold, but never with amalgam. Tin is the best material in a majority of cases. I must not be understood as condemning the use of amalgam entirely, as I think it an excellent material in its proper place.

*Dr. Green*—I fill the teeth Dr. Noyes has just mentioned, up to fourteen years, with cements. The average durability of such fillings is about four years.

*Dr. Stevens*—I am opposed to the use of cocaine. From my observation and reading I should as readily consent to use chloroform for an anæsthetic. In one or two cases death has resulted from the use of the two per cent solution of cocaine. I do not believe in employing corundum disks, but use a great many of sandpaper. Broken pieces of files may be used to advantage as matrices, instead of the patented and advertised articles.

*Dr. Cady*—If Dr. Stevens will try the Brophy matrix, he will understand its great advantages over the office-made instrument. Until about six months ago I was, after having tried a number, opposed to the use of any form of matrix, but at the earnest solicitation of a friend whose operative abilities I recognize, I tried the little instrument devised by Dr. Brophy, and was much pleased with it. I am satisfied that its use will, in many cases, save a vast deal of time and nervous strain to both patient and operator. The method of using gold and tin combined at the cervical margin, where the cavity extends to or below the gum, is admirable. The first third of the cavity I always fill with non-cohesive gold, and the finishing is done with No. 60, cohesive. Sandpaper disks are valuable adjuncts to the operating case, but I prefer those of emery-cloth. These, when moistened with oil, cut faster and with less pain to the patient from heat generated by the rapid motion. In addition to this, they are more pliable, and can be directed with a properly-shaped instrument into depressions and irregularities of surface that no other disk will reach.

*Dr. Newkirk*—I find most of the finishing tapes on the market too wide for my purposes. The narrow tape, to be obtained at dry goods stores, should be employed. I use the scalers of Dr. Cushing's set to follow out and cut off thin laminæ of gold at the cervical edge.

*Dr. Stevens*—Sandpaper disks, properly used, are better than tape, or anything else, for finishing the cervical edge.

*Dr. Harlan*—Many fail in adjusting the rubber-dam, because the mouth and teeth are not first thoroughly cleansed. This should be attended to first of all. Others use the ligature too thick, and without scraping. I do not believe in the indiscriminate use of matrices, but when they are used the same care in all steps of the operation should be observed as when they are not. Hydraulic gutta-percha is the best material for wedging. In the young there is great danger to the pulp and the peridental membrane in rapid separation. The separator, in any form, should be used very judiciously.

*Dr. Woolley*—I believe the matrix is a valuable instrument in its place, but the laws governing its use should be recognized.

*Dr. Brophy*—If tin and gold, or tin alone is used at the cervical margin, there will be no occasion to use at that point either disks or strips in finishing. All the polishing necessary can be done with the burnisher in most cases. If it is desirable to use anything more, Dr. Parmly Brown's strips, used with corundum mixed with glycerine to a paste of the consistency of honey, will be found superior to all else. Sandpaper disks and tape bear no comparison. The testimony of a gentleman concerning an instrument he has never used is of little value. Matrices are invaluable in many cases. They are frequently so in using amalgam, and more often with gold. In using them no retaining pits should be made, nor any groove made at the cervical margin. The floor of the cavity should be left smooth, and gently sloping toward the gum from the interior. Anchorages should be made in the lateral walls, some distance from the bottom, and sufficiently far from the top to preclude weakening the masticating surface. I hope the old methods of preparing and filling cavities are past, not only on our own account, but for the benefit of the community at large. It is impossible to use a piece of broken file for a matrix with success. With such an instrument we have a flat, unyielding surface, that will not conform to the tooth. Fillings made by its use are invariably flat and without contour, and the cervical margin is always a point of uncertainty. I agree with Dr. Harlan, that the indiscriminate use of matrices is wrong, but so is the indiscriminate use of almost any instrument that can be mentioned. It is best to start the separating process slowly. In certain cases, nothing better has been devised than Perry's Separator, and this is specially true in country practice, where patients come many miles. Use the instrument carefully, turning slowly until slight pressure is obtained. Then separate, stopping often to turn the screw up a little more, until the space desired is made. I want to urge the use of soft gold, or better still, tin and gold, at the cervical margin and for the first third of cavities. Injudicious use of the ligature is often the cause of pyorrhœa alveolaris, from forcing the gum back and allowing the formation of salivary calculus.

*Dr. Black*—I want to utter a word of warning regarding the use of the matrix, when the bottom of the cavity is bowl-shaped. The gold, in such cases, will roll up, or turn one side, and the filling will be insecure from the start. I think it necessary to make the bottom of the cavity angular in some cases, to prevent this. Failure in contouring approximate surfaces of molars is the cause of more complaints among our patients than anything else, perhaps. In cases where decay has allowed the teeth to come together too closely, we should wedge a great deal, but slowly, because we must wedge so much. The cavities should often be filled temporarily, in order to wedge as desired. After the considerable space necessary is obtained, a matrix should be adjusted which will fit closely at the cervical margin. This must be loosened or removed at the proper point, and the filling contoured and finished.

*Dr. Allport*—I must condemn the general use of matrices, while recognizing the value of their occasional employment. I do not believe a good operation can be made with a band matrix when the cutting edge is larger than the neck of the tooth. A perfect fit cannot here be made at the cervical edge. One use to which the wedge may be put is to hold a tooth still. I am glad to hear non-cohesive gold favorably mentioned. I have advocated its use with broad instruments for twenty years. I have seen hundreds of teeth spoiled by improper preparation of their cavities, and the subsequent breaking away of sharp or thin edges in filling. I am in the habit of polishing the edges of cavities with pumice stone. If proper care is exercised in preparing and filling a tooth, but little finishing will be required. I have little need of retaining points. In concluding, let me urge the use of large instruments.

EVENING SESSION—PRACTICAL THERAPEUTICS.

The discussion of Dr. Harlan's paper, which had been continued from the session of Wednesday evening, was resumed.

*Dr. Freeman*—Dentists should at least try new remedies and learn the truth of the claims advanced in their favor. Many fail to obtain good results from peroxide of hydrogen.

*Dr. Gardiner*—I am not surprised at this, as dentists are not sufficiently careful in purchasing, and buy in too small quantities. Marsh's and Tromsdorf's preparations of $H_2O_2$ are both good. Glycozone is an excellent remedy.

*Dr. Reed*—I think one of the causes of complaint about peroxide of hydrogen is that the rubber-dam is not always adjusted before the remedy is applied.

*Dr. Harlan*—Peroxide of hydrogen is very useful as a detecter of pus in the Antrum of Highmore. Iodol is stimulant, without being an irritant. The effect of cocaine applied to the mucous membrane is to produce anæmia and paralysis of the vaso-motor nerves.

*Dr. Gardiner*—Warm water should always be injected freely into the antrum before using $H_2O_2$. In one case where I neglected this precaution, the effervescence caused by the remedy coming in contact with pus nearly suffocated the patient.

*Dr. Black*—I want to reiterate the necessity of applying the rubber-dam in all cases where the pulp-chamber is exposed. With our present knowledge of microbes we can feel assured that success will attend our efforts if proper antiseptic precautions are taken. Since we say positively that pus will not be formed without microbes, we should bear constantly in mind the importance of necessary precautions to hold the latter at a safe distance.

Dr. Morrison read his paper—

OPERATIVE DENTISTRY AS APPLIED TO DECIDUOUS TEETH.

The following is a rather brief abstract: It should be our aim to save all of the crowns in their natural shape and position, and by proper care to keep them thoroughly cleansed. Simple, regular-shaped cavities can be filled with gold, in cylindrical form preferably, retaining pits being seldom necessary or admissible. Continuous malleting or the use of much force should be avoided. Amalgam has a wide range of usefulness. It can be used for simple cavities, and for the larger and more complicated it has no superior. Heroic excavating should not be practiced, as it often endangers the life of the pulp. Leave the soft dentine, if necessary, over the pulp tissues, securing slight undercuts only—merely enough to prevent the fillings from being lifted from their beds. Exposed pulps should be capped with gutta-percha dissolved in chloroform, or phosphate of zinc, where there is the slightest hope of retaining their vitality. When dead, remove all débris from the chamber and canals, and disinfect with wood creosote. Fill the roots with the gutta-percha solution, and the cavity with amalgam, being careful to polish the latter at a subsequent sitting.

Cements and gutta-percha are of too uncertain action, and too temporary in character for even deciduous teeth, gutta-percha swelling out of the cavity and the cements being too readily washed away.

About the time the centrals are erupted there is considerable pressure on the approximal surfaces, and when decay at these points is neglected the surfaces drop quite into each other. In this instance I wedge them open with cotton for a few days, and if the cavities are of such shape that individual fillings cannot be made so as to retain themselves in each tooth separately, I would block the entire space full, reaching from the undercut of the enamel of one crown across to the undercut in the other tooth, leaving the whole mass of the filling bridged from one tooth to the other, thus vastly improving the power of mastication, and keeping the arch braced to assist in the development of the second teeth, which is taking place below.

I rarely extract devitalized roots, but file and grind them smooth, a little short of occlusion. Educate the parent as well as the child, so that when they bring the first permanent molar decayed almost beyond recognition, they will not say, "I thought it was a baby tooth."

Too much sweets and insufficient diet are the causes of such imperfectly organized teeth, and the approximal pressure on the vitreous enamel, surrounded by vicious secretions, greatly accelerates their decay. Teach parents how to forcibly oxygenate their blood with the purest out-door air they can get, filling the lungs to their fullest extent, holding it a moment and then contracting the chest muscles upon them, thus forcing the air into the remote territory which is not used on ordinary occasions. Use boiled water to drink. Whether it be from hydrant, cistern or well, by heating to the boiling point we destroy the organic life, which could not be other than harmful. Use sulphur as a dentifrice to destroy any parasitical life. By this line of treatment, in a few generations irregularities of the permanent teeth would be of very rare occurrence. Let all our efforts for these teeth be toward their preservation to the very last hour the individual can make any use of them, and let it be our aim to impress upon parents the necessity of proper care and attention upon their part. The value of deciduous teeth has never been properly appreciated by parents. I regard them as being just as important in their sphere as permanent teeth are in theirs.

*Dr. K. B. Davis*—Amalgam, tin, and phosphate of zinc may be used in those teeth, but gold—never. Injudicious extraction causes irregularities in the permanent teeth. Parents are often surprised to know that the first permanent molar belongs to the second set.

*Dr. Brophy*—I believe the greatest objection to extracting these teeth is that it causes too early eruption of the permanent successors. I have many models which substantiate this position. The permanent teeth, if too early erupted, are not sufficiently calcified to withstand those influences that bring about decay. Besides, it leads to irregularities and mal-position. On the other hand, we have trouble resulting from too long retention of the temporary teeth. This subject is a science in itself.

*Dr. Newkirk*—Nature's plan is for each temporary tooth to remain in place until the time comes for the appearance of its successor. The prevention of toothache is important. Pain banishes sleep. Sleeplessness leads to nervousness, and this to indigestion and disorders of the whole alimentary tract. A tooth with a dead pulp may be succeeded by an abscess. Such a tooth is sore, and in consequence the child cannot masticate, but bolts its food, and the evil results of indigestion follow. I am doubtful about the advisability of bridging a filling from one tooth to another. Such an operation would interfere with the natural movement of the teeth, and the fillings would not probably be permanent, unless the teeth and anchorage were unusually strong.

*Dr. Ottofy*—I object to the bridging here mentioned, on account of the difference of time in shedding the teeth. Teeth usually come in earlier on the right side than on the left.

*Dr. Harlan*—I have had the observation of a rather large family of my own, and think the second temporary molar seldom erupts before the twenty-fourth month, and sometimes as late as the thirty-third. Dr. Morrison is mistaken in supposing that sulphur will destroy parasitical life. Powdered sulphur is inert, and not disinfectant.

*Dr. Kester*—Tin and gold, or tin alone, are excellent for use in the crowns of deciduous teeth. I like red gutta-percha also, and use it for bridging.

*Dr. Black*—Premature extraction of temporary teeth begets an irregularity of eruption, and teeth may come in too late as well as too early. The enamel in the permanent set is sufficiently calcified in all cases when the teeth are erupted, and the softening mentioned by a speaker is due to a diseased condition of the roots of the temporary teeth which occupied the space above it. An abscess on the root of a temporary tooth affects the building of enamel on its successor, and makes it faulty in structure and form. There is likely to be a contraction of the arch from early extraction of temporary teeth, but this does not necessarily follow. An arch is sometimes expanded by the advance of the permanent teeth. The alveolar process of the temporary teeth is never the alveolar process of the permanent ones. I want to say that I do not like bridging with amalgam. There is some excuse for it with gutta-percha or cement. A good plan in many cases is to make a little platinum plate, with a pin soldered to the lower side for anchorage, which is pressed down into the warm gutta-percha and left to bear the brunt of mastication.

FRIDAY MORNING.

Dr. T. W. Brophy read a paper on the "Diagnosis of Oral Tumors." In the broadest sense a tumor is a swelling or puffing up of the tissues, and may manifest itself in various forms. It has been described as a local limited enlargement taking place at any part of the body, and consisting in its substance of a new outgrowth of tissue which has no physiological purpose in its growth. Our knowledge of the true characteristics of tumors has been acquired by means of the microscope, and a knowledge of minute anatomy is indispensable to the student of pathology, as he must be able to detect the changes that take place in the tissues as a result of perverted nutrition. Tumors are studied under two heads—histological and clinical. Their etiology, to a great extent, is still wrapped in obscurity. Conheim has advanced an ingenious theory, saying: "There may be produced in an early stage of embryonal development more cells than are necessary for the construction of a definite part, so that a certain number of cells remain superfluous. Their number may be small, but they possess great proliferating power on account of their embryonal nature." According to this author these supernumerary embryonic cells are the origin of all subsequent growths, in the form of tumor during life, but his views are not generally subscribed to.

For surgical consideration, tumors are divided into two great classes—benign and malignant. While experience enables the surgeon to correctly diagnose tumors in nearly every instance, the microscope should be employed to settle beyond question the character of the growths. The method of diagnosis should be that of exclusion; *i. e.*, taking up the various conditions which may lead to the formation of a tumor, and, step by step, by both subjective and objective signs, and by manipulation, determine the character of the growth.

A patient presents himself, having a tumor over the anterior surface of a superior maxilla, and a marked prominence is observed. What is the cause and character of this abnormality? It may be active pericementitis, an alveolar abscess, an odontocele, an angioma, a dental exostosis, an osteoma, a fibroma, a distention of the walls of the antrum in consequence of an accumulation of fluid, a cancerous growth, or any other form of oral tumor. In making a diagnosis we should proceed methodically, and consider with care the exciting and predisposing causes, keeping in mind the anatomy of the parts. Is the enlargement due to pericementitis? The teeth are sound and not sensitive to pressure. None seem longer than the others, and occlusion is not painful. No, the enlargement is not due to pericementitis. Is it the result of alveolar abscess? The arch contains its full complement of teeth, and pulpless ones are not present. Fluctuation is not observed, and the needle does not evacuate pus. The swelling is not caused by an alveolar abscess. Is it an odontocele? This, as its name implies, requires an unerupted tooth for its nucleus. As before remarked, the dention is unbroken. We must remember, however, that an odontocele may have for its origin a supernumerary tooth. On introducing a sharp probe or exploring needle we do not observe the characteristic ring of the enamel which follows the stroke of steel. A tooth is not in the growth. It is not an odontocele. Have we before us an angioma? An incision causes but ordinary hemorrhage, while a vascular tumor bleeds profusely when its walls are divided. It is not, then, an angioma, a dental exostosis, or an osteoma. The sharp probe readily enters its substance, showing it is not an osseous growth. A fibroma is indurated and sometimes has the feel of bone. The probe enters it with difficulty, since the mass is tough and apparently cartilaginous in structure. We have not here, then, a fibroma nor an epulic tumor, which has many characteristics of the former. Is this swelling due to a bulging of the antral walls in consequence of an abscess therein ? What are the symptoms of abscess of the antrum ? Dull, steady pain, a sense of fullness on the affected side, and often a diseased tooth as the origin of the lesion. When the natural opening between the nasal cavity and the antrum is closed, the osseous walls may become thin by absorption and bulge out. When the natural opening is not closed, the fluid escapes into the nose. Fluid has not found its way through the anterior wall of the antrum to cause the bulging. The antrum is not diseased.

We might in this manner consider all explainable tumors, and exclude them all. What, then, shall our diagnosis be ? The origin of the growth is not found, and we cannot account for the presence of the tumor. A growth which cannot be proved benign should be considered a cancer, and treated as such. The line of treatment is prompt and thorough removal by a surgical operation.

*Dr. Black*—The subject of diagnosis is one of great difficulty to tell or write about. Little can be learned from the simple description of these growths. Observation is absolutely necessary for a thorough knowledge of the subject. The method of diagnosis by exclusion is very good, but we must thoroughly examine and re-examine all points of the case. The sharp corners of teeth are often the prime factors in the development of epithelioma. Epithelioma, occurring about the mouth and lips, outnumber all other causes combined. This subject is not properly understood by many of the profession. I remember a case in which through faulty diagnosis a large section of healthy alveolar process was removed. It is better, however, to cut away many times when unnecessary than allow one case of epithelioma to go too far. Epithelioma travels by way of the lymphatics, and after these are once attacked the patient is lost. Being transplanted through the glands to other parts of the anatomy, you will understand the importance of operating early. This manner of being carried from one part to another is general, and does not relate to tumors of the mouth alone. In cancer of the stomach, for instance, the liver is never free from cancerous deposits, carried there in the blood-stream. Cancer of the tongue and lips is more dangerous than those of immovable parts.

*Dr. Cady*—It is important that we be able to diagnose these tumors ourselves, and not compelled to rely upon the judgment or ask the advice of physicians. Especially is this essential in a country practice. During the few years that I practiced in a small city, the importance of this knowledge was brought forcibly to my notice. One case that came under my care was a sarcoma of the superior maxillary, and though I urged the necessity of an immediate operation, the family physician counseled otherwise, and advised the use of listerine and myrrh, which he said would speedily bring about resolution. Fancy listerine and myrrh as a treatment for sarcoma! When at last the patient was operated upon it was found necessary to remove all the bone in the right side of the face from the median line, including the maxillary and malar bones and the floor of the orbit, an operation requiring over two hours. And yet simple antiseptic and astringent treatment was expected to effect a cure in such a case. The patient lived about a year after the operation, when the disease made its appearance in the glands of the neck and he speedily died, even listerine and myrrh being powerless to save him. Another case was sent me by a physician, with instructions to remove a superior molar, which, from being incrusted with calculus, was supposed to have caused "a sore spot which wouldn't heal while the tooth was there." A very brief examination convinced me that the "sore spot" was nothing less than a typical case of carcinoma, and as the patient was fifty years of age, and stated that his father had died of cancer, the prognosis was decidedly unfavorable. Imagine the pathological acumen of a man who pompously designates as a "sore" a malignant ulcer of this nature, with its characteristic appearance, which is almost unmistakable, and the fine way which he coolly directs the removal of a tooth as a cure for the most painful constitutional disease that flesh is heir to. I repeat, we should have a knowledge of these tumors, which places us above the necessity of consulting medical practitioners when a case presents for diagnosis.

*Dr. Brophy*—It is important to go far beyond the limits of this disease in operating, rather than stop a trifle short of it. I present for your examination a dermoid cyst, removed from the ovary of a woman sixty years of age. As you will observe, it contains four teeth and some hair.

Dr. L. L. Davis read a paper upon—

THE USE OF THE MICROSCOPE IN PROGRESSIVE DENTISTRY.

Though long regarded as a mere toy, the microscope now takes place among the useful and important adjuncts of medical and dental practice. It has done more than any other one thing to advance the progress of science, unveiling the mysteries of nature and opening a new world unto the student, and its present importance warrants the assertion that it is to become one of the prime educators of rising generations. It is but a short time since the study of microscopy was introduced into the curriculum of dental and medical schools, yet of such importance has this work proved itself that no college dare slight it at the present time. It is not expected that the student will become an expert in histological work during the short time spent in college, any more than it is supposed he will become skillful in the other branches of study, for skill and expertness are only acquired by experience, but the foundation should be laid so efficiently that after-life will prove the value of such training. Faith is not, as many suppose, the most essential qualification for the progress of this study. Let a man doubt every theory he reads till his own eyes and reason have compelled him to give assent to its truth, or show its utter falsity. For the young man there is no field so wide in which to carve out name and fame, and in no other line of intellectual development will be found so fascinating and pleasant an occupation.

*Dr. Gilmer*—It is essential that all should have at least a knowledge of practical microscopical matters. A professional man should know something of all things, and all things of some one thing. He should be as familiar with the formation of a tooth as with its external appearance, and the student learns more of a tooth's structure from the microscope than from any number of didactic lectures and illustrations. I am glad this subject is taking root among the members of the Illinois State Dental Society, and hope it will increase till all are practical microscopists.

